# Feasibility and outcome of partial open surgical fenestrated stent graft explantation, radical debridement, and in situ reconstruction for late graft infection

**DOI:** 10.1016/j.jvscit.2023.101175

**Published:** 2023-04-23

**Authors:** Jesse Manunga, Christopher Pedersen, Benjamin Selle, Elliot Stephenson, Nedaa Skeik

**Affiliations:** a Section of Vascular and Endovascular Surgery, Minneapolis Heart Institute at Abbott Northwestern Hospital, Minneapolis, MN; b Minneapolis Heart Institute Foundation, Minneapolis, MN

**Keywords:** Complex aortic aneurysm, Fenestrated stent graft infection, Fenestrated stent graft explantation, Partial fenestrated stent graft explantation for infection

## Abstract

Aortic stent graft infection is a rare, but potentially lethal, complication of endovascular aortic aneurysm repair. Definitive treatment is complete stent graft explanation with in-line or extra-anatomical reconstruction. However, several factors can render such an operation unsafe, including the patient's overall fitness for surgery and partial incorporation of graft with a resulting robust inflammatory process, especially around the visceral vessels. We present the case of a 74-year-old man with a history of an infected fenestrated stent graft that was managed with partial explantation, wide debridement, and in situ reconstruction using a rifampin-soaked graft and a 360° omental wrap with good results.

The use of fenestrated and branched endografts for repair of complex aortic aneurysms continues to gain worldwide acceptance owing to the technique's minimally invasive nature and shorter convalescence compared with open surgical repair.^1^, ^2^, ^3^ One of the most feared complications of endovascular abdominal aneurysm repair and thoracic aortic aneurysm repair is stent graft infection, occurring at a rate of 0.2% to 5%.^4^, ^5^, ^6^, ^7^

Medical management with antibiotics alone or combined with percutaneous aneurysm sac drainage has been offered with various degrees of success.^8^^,^^9^ However, definitive treatment is complete stent graft explanation with extra-anatomic or in-line reconstruction using a homograft, autogenous veins, or antibiotic-soaked prosthetic grafts.^5^ The choice of treatment offered is often dictated by a number of factors, including the patient's overall fitness for surgery. Stent graft explantation has a reported early mortality of ≤25% and high complication rates.^10^^,^^11^ Although currently unknown, it is safe to speculate that these risks are higher for patients undergoing fenestrated stent graft (fenestrated endovascular aortic aneurysm repair [FEVAR]) explantation.

We present the case of a 74-year-old man with history of FEVAR for a juxtarenal aortic aneurysm. He had presented to an outside institution with constitutional symptoms and found to have an infected stent graft. He underwent partial explantation and reconstruction using a rifampin-soaked Dacron graft with good clinical results and complete resolution of infection found at the 3- and 6-month follow-up examinations and fluorine-18 fluorodeoxyglucose positron emission tomography (PET) scans. The patient provided written informed consent for the report of his case details and imaging studies.

## Case report

**Fig 1:**
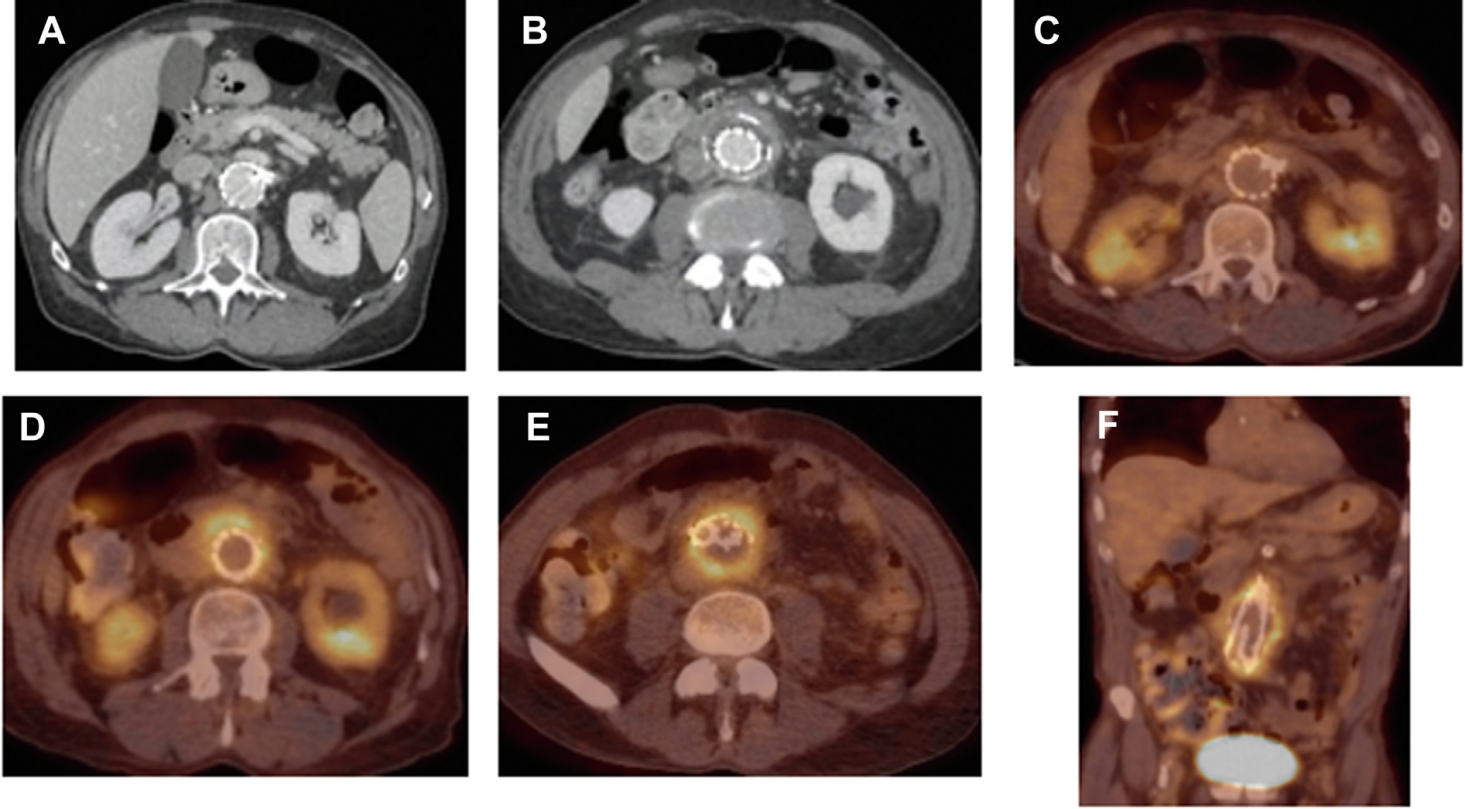
Preoperative computed tomography angiography and positron emission tomography (PET). Note the well-incorporated graft in the paravisceral segment **(A)** and thickening and/or inflammatory changes of the infrarenal aorta concerning for infection or aortitis **(B)**. Similarly, note that the paravisceral aorta is free of any uptake on the preoperative PET scan in the paravisceral aorta **(C)** but the significant uptake in the infrarenal aorta was limited to the aortic sac, sparing the endograft itself **(D-F)**.

A 74-year-old man with cardiovascular risk factors significant for hypertension, hyperlipidemia, chronic obstructive pulmonary disease, congestive heart failure, ongoing tobacco dependence, and 5.6-cm juxtarenal aortic aneurysm underwent three-vessel FEVAR using a Zenith fenestrated stent graft (Cook Medical Inc) with stenting of the superior mesenteric artery and bilateral renal arteries (RAs) in 2015 with good results. He presented to an outside institution 7 years later with complaints of abdominal and back pain, poor appetite, malaise, night sweats, and failure to thrive. The laboratory study results were significant for leukocytosis and hyponatremia. Computed tomography angiography revealed a remodeled aneurysm sac, thickening of the aneurysm wall, and possible periaortic fluid collection that was concerning for aortitis and/or infection (Fig 1, *A* and *B*). Broad-spectrum antibiotics were started, and he was transferred to our institution for further management. His blood cultures showed no growth. The findings from an extensive infectious and inflammatory workup led by infectious disease and vascular medicine at our institution, including for syphilis and human immunodeficiency virus, and blood cultures were negative. Rheumatologic serology tests, including for antinuclear antibody, antineutrophil cytoplasmic antibodies, complements, cyclic citrullinated peptide, and immunoglobulin G, were all normal.

**Fig 2:**
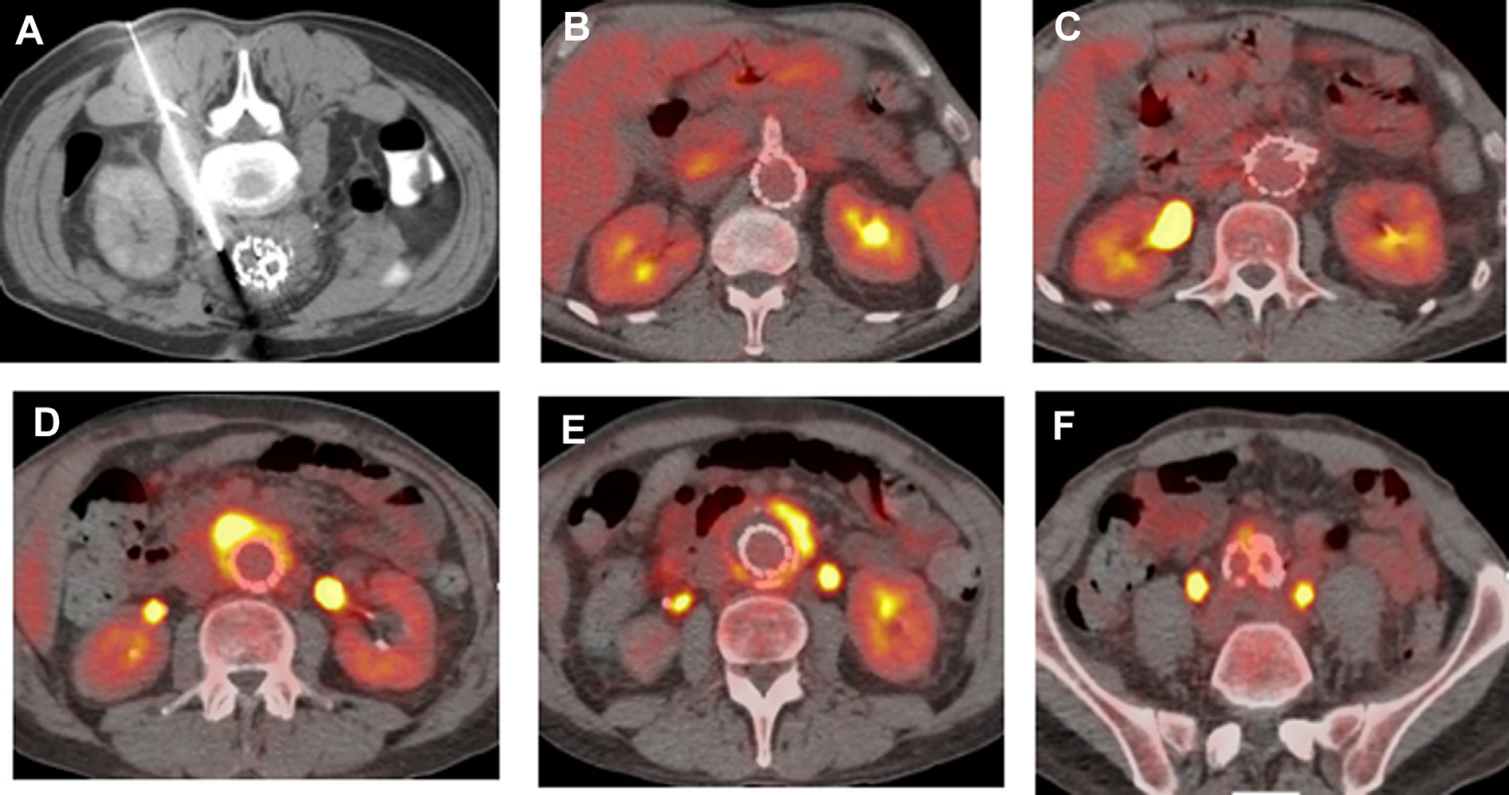
**A,** Computed tomography-guided aspiration of the aneurysm sac content. Cultures did not show any growth, which was not surprising, because the patient had been receiving broad-spectrum antibiotics for weeks before this procedure. PET continued to show no evidence of infection in the paravisceral aorta **(B,C)** but significant uptake in the infrarenal aorta **(D-F)**.

PET revealed increased, noncircumferential, uptake limited to the infrarenal aortic sac tissue suggestive of infection (Fig 1, C-F). Subsequent computed tomography-guided aspiration of the periaortic fluid returned milky fluid that did not grow any bacteria (Fig 2, A). The patient was offered stent graft explanation but opted for a trial of intravenous antibiotics, with cefepime, vancomycin, and metronidazole. Intolerant of broad-spectrum antibiotics and still symptomatic, he returned 1 month later for reevaluation. The laboratory test results demonstrated neutropenia (white blood cell count 1.6) and a persistently elevated sedimentation rate but improved C-reactive protein. A repeat PET scan showed continued uptake in the infrarenal aorta (Fig 2, B-F), and he agreed to undergo graft explantation.

## Operative details

**Fig 3:**
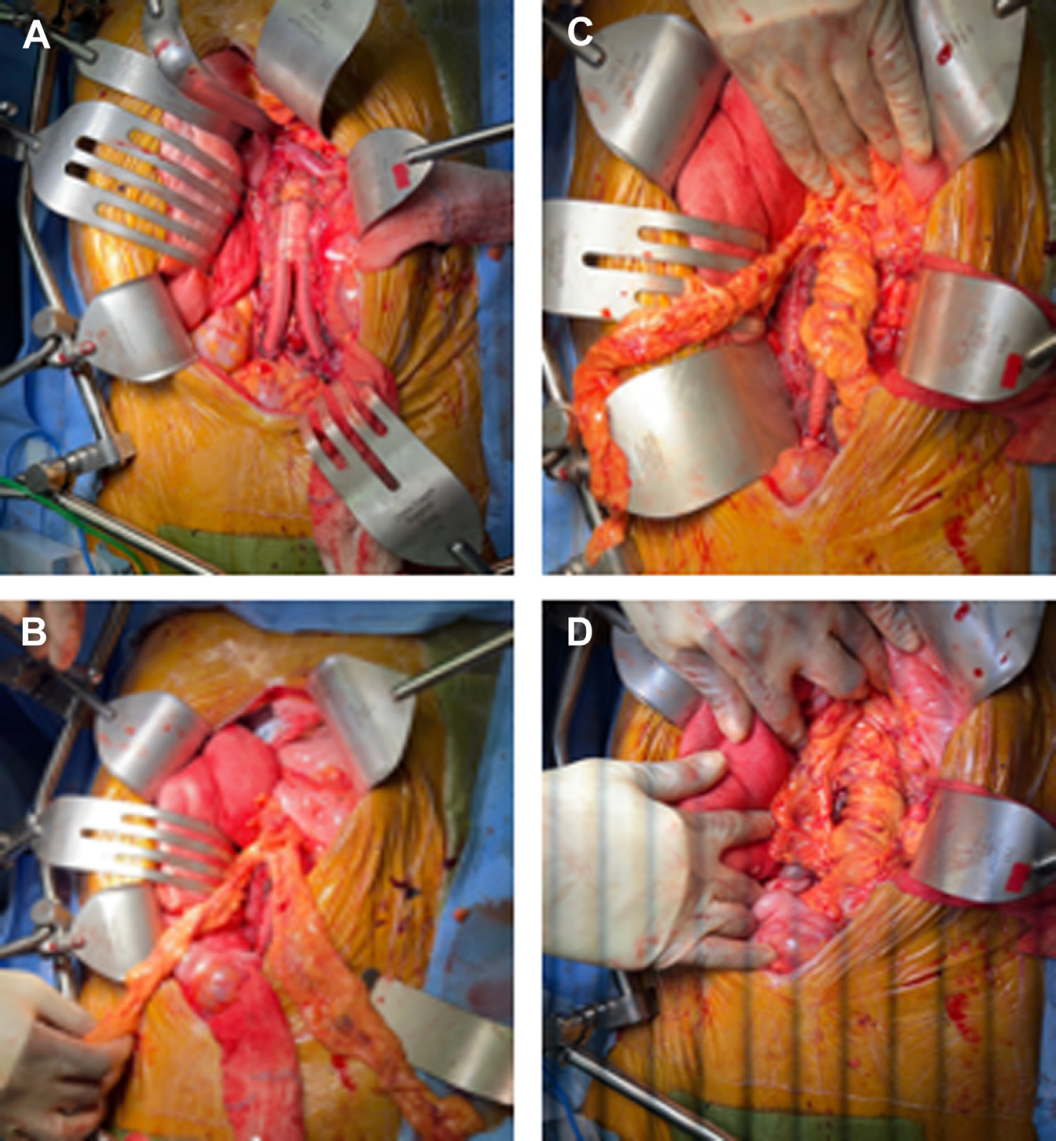
Intraoperative photographs. Note the radical debridement of the infrarenal aortic tissue to healthy and well-incorporated tissue. The rifampin-soaked Dacron graft is sutured to the fenestrated cuff, and the entire universal bifurcated device has been explanted **(A)**. Omental “tongues” have been created **(B)** and used to perform a 360° wrap of the newly placed bifurcated rifampin-soaked Dacron graft **(C,D)**.

**Fig 4:**
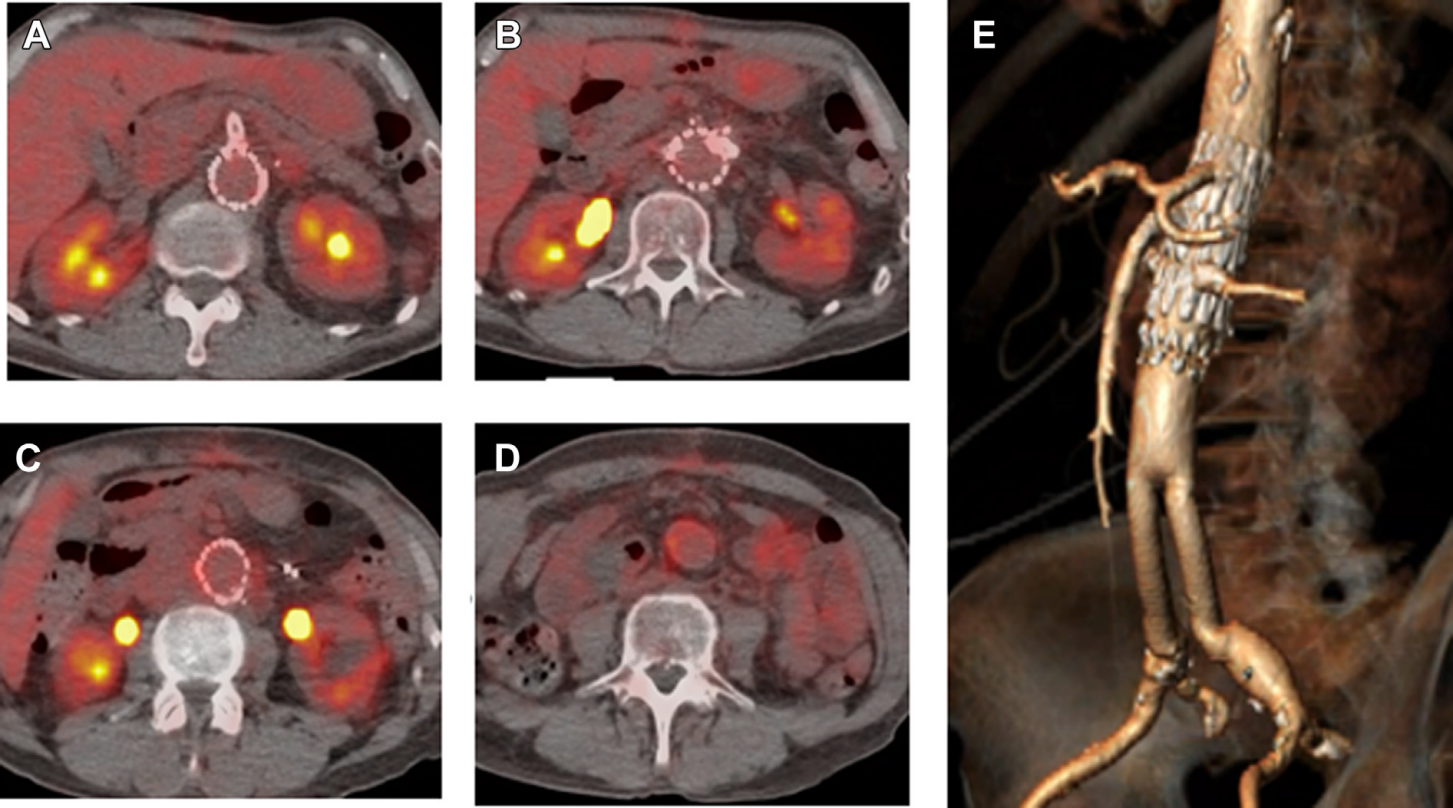
Postoperative positron emission tomography (PET) scans **(A-D)** showing no uptake and three-dimensional reconstruction computed tomography angiogram showing the residual fenestrated cuff and bifurcated device **(E)**.

The procedure was performed via a midline transperitoneal approach. An attempt was made to perform a left medial visceral rotation.^12^ However, severe inflammation was encountered around the visceral arteries and the stent graft appeared well-incorporated in this region, making dissection unsafe. The supraceliac aorta and celiac artery were exposed and prepared for clamping. The patient was systemically heparinized, and the iliac arteries and aorta were clamped at the diaphragmatic hiatus for 10 minutes. The aortic sac was open longitudinally, and all unincorporated tissue was debrided down to the anterior spinal ligament before transferring the clamp to the infrarenal position. The universal bifurcated device was explanted, and the fenestrated cuff was partially excised, leaving a sewing ring in the infrarenal aorta. The periaortic fluid, debrided tissue, and graft were sent to the laboratory for culture. The infrarenal aorta was reconstructed with an 18-mm × 9-mm rifampin-soaked Dacron graft with a 360° omental wrap (Fig 3). No drains were left in place. The estimated blood loss was 2 L. The patient was discharged home on postoperative day 8 with a 6-week course of ertapenem, followed by amoxicillin and clavulanic acid for 3 months. CTA and PET obtained at 3 and 6 months postoperatively showed no evidence of infection (Fig 4). At the last follow-up, the patient remained asymptomatic and has continued to gain weight.

## Discussion

To the best of our knowledge, four cases of FEVAR explantation have been reported in the English literature, making ours the fifth case. Three of these were for infection^13^, ^14^, ^15^ and one for a recalcitrant type II endoleak.^16^ An additional patient was treated medically using antibiotics, with good results.^17^ Unlike our patient, all three patients offered explantation had a secondary aortoenteric fistula (SAEF). In the first patient, the aorta was clamped above the RAs, the bridging stents were crushed and flattened, the entire fenestrated device and bridging stents were removed, and in situ reconstruction was performed in the infrarenal aorta.^15^ The second patient underwent complete graft explantation with extensive in situ reconstruction using an autologous vein graft with bypass to the RAs. The procedure was complicated by bowel ischemia requiring resection, and the patient died of multisystem organ failure.^13^ The third patient underwent explantation with superior mesenteric artery and bilateral RA reconstruction using a Intergard Synergy antimicrobial graft (Getinge) and did well (Table).^14^

**Table:** Literature review and outcomes of patients undergoing fenestrated stent graft explantation in the English literature

Investigator	Age, years; sex	LOTTE, months	Reason for explantation	Conduit	Partial vs total	Complication	HLOS	Follow-up, months
Terry et al,^13^ 2017	72; Male	10	Infection	Superficial femoral and great saphenous vein	Total	Two revisions for bleeding, bowel ischemia with subtotal colectomy and small bowel resection	Died POD 3	NA
Nordanstig et al,^15^ 2019	66; Male	26	Infection	Femoral vein (NAIS)	Total	NR	3 Weeks	24
Caradu et al,^14^ 2020	67; Male	12	Infection	Bifurcated antimicrobial Dacron graft	Total	*Stenotrophomonas pneumonia*, acute occlusion of left renal bypass at 12 months postoperatively	30 Days	12
Steadman et al,^16^ 2022	77; Male	60	Recalcitrant type II endoleak	Polyester graft	Partial	SMA stent compression requiring balloon angioplasty intraoperatively	6 Days	48
Present study	74; Male	84	Infection	Rifampin-soaked Dacron graft	Partial	None	8 Days	6

*HLOS,* Hospital length of stay; *LOTTE,* length of time to explantation; *NAIS,* neoaortoiliac system; *NR,* not reported; *POD,* postoperative day; *SMA,* superior mesenteric artery.

For our patient, we had planned for complete stent graft explantation. Our plan changed after realizing that the tissue above the RAs appeared uninvolved in the infectious process. Also, compared with the infrarenal device, the suprarenal stent graft was well incorporated, and no pus, abscess, or any SAEF was found. This was consistent with the PET findings (Figs 1, *C-F* and 2, *B-F*). Our decision was guided by reports from the literature showing no reinfection in patients who had undergone partial stent graft explanation, wide infected tissue debridement, in situ reconstruction, and 360° omentum wrapping.^4^^,^^18^

It is reasonable to argue that an autogenous or a homograft should have been the conduit of choice. The decision to use a rifampin-soaked Dacron graft was because (1) unlike “early” infections, “late” graft infections without obvious SAEF usually result from less virulent pathogens; (2) no pus or abscess was found during the operation; (3) the cultures were negative; and (4) rifampin-soaked Dacron grafts have been proved effective, even in cases of pseudomonas as long as the infected tissue is debrided and the graft is wrapped 360° in omentum, as was it was for our patient (Fig 3).^18^ Graft infection recurrence is always a concern and has been reported to occur as early as 2 weeks and as late as 6 years after the initial explantation.^19^ As such, close and lifelong follow-up is imperative.

## Conclusions

Partial FEVAR explantation with wide debridement of all infected tissue, followed by in situ reconstruction with a rifampin-soaked Dacron graft and 360° omental wrap, appears to be a safe alternative to complete stent graft explantation for patients with no pus or abscess and a well-incorporated graft. Patients should be followed up for life and advised regarding the risk of recurrence.

## References

[1] Kristmundsson T., Sonesson B., Dias N., Torqvist P., Malina M., Resh T. (2014). Outcomes of fenestrated endovascular repair of juxtarenal aortic aneurysm. J Vasc Surg.

[2] Oderich G., Greenberg R., Farber M., Lyden S., Sanchez L., Fairman R. (2014). Result of the United States multicenter prospective study evaluating the Zenith fenestrated endovascular graft for treatment of juxtarenal abdominal aortic aneurysms. J Vasc Surg.

[3] Gallitto E., Faggioli G., Pini R., Mascoli C., Ancetti S., Fenelli C. (2019). Endovascular repair of thoraco-abdominal aortic aneurysms by fenestrated and branched endograft. Eur J Cardiothorac Surg.

[4] Fatima J., Duncan A.A., de Grandis E., Oderich G.S., Karla M., Gloviczki P. (2013). Treatment strategies and outcomes in patients with infected aortic endografts. J Vasc Surg.

[5] Smeds M.R., Duncan A.A., Harlander-locke M.P., Lawrence P.F., Lyden S., Fatima J. (2016). Treatment and outcomes of aortic endograft infection. J Vasc Surg.

[6] Capocccia L., Mestre G., Riambau V. (2014). Current technology for the treatment of infection following abdominal aortic aneurysm (AAA) fixation by endovascular repair (EVAR). J Cardiovasc Surg (Torino).

[7] Laser A., Baker N., Rectenwald J., Eliason J.L., Criado-Pallares E., Upchurch G.R. (2011). Graft infection after endovascular abdominal aortic aneurysm repair. J Vasc Surg.

[8] Katz B.H., Black R.A., Colley D.P. (1987). CT-guided needle aspiration of periaortic collection. J Vasc Surg.

[9] Blanch M., Berjon J., Vila R., Simeon J.M., Romera A., Riera S. (2010). The management of aortic stent-graft infection: endograft removal versus conservative management. Ann Vasc Surg.

[10] Chervu A., Moore W.S., Gelabert H.A., Colburn M.D., Chvapil M. (1991). Prevention of graft infection by use of prostheses bonded with a rifampin/collagen release system. J Vasc Surg.

[11] Chaufour X., Gaudric J., Goueffic Y., Khodja R.H., Feugier P., Malikov S. (2017). A multicenter experience with infected abdominal endograft explantation. J Vasc Surg.

[12] Reilly L.M., Ramos T.K., Murray S.P., Cheng S.W.K., Stoney R.J. (1994). Optimal exposure of the proximal abdominal aorta: a critical appraisal of transabdominal medial visceral rotation. J Vasc Surg.

[13] Terry C., Houthoofd S., Maleux G., Fourneau I. (2017). Explantation of an infected fenestrated abdominal endograft with autologous venous reconstruction. EJVES Short Rep.

[14] Caradu C., Dinclaux V.V., Lakhlifi E., Dubuisson V., Ducasse E., Berard X. (2020). Surgical explantation of a fenestrated endovascular abdominal aortic aneurysm repair device complicated by aorto-enteric fistula. EJVES Vasc Forum.

[15] Nordanstig J., Torngren K., Smidfelt K., Roos H., Langenskiold M. (2019). Deep femoral vein reconstruction of the abdominal aortic and adaptation of the neo-aortoiliac system bypass technique in an endovascular era. Vasc Endovascular Surg.

[16] Steadman J.A., Mendes B.C., Oderich G.S. (2022). Technique of partial open surgical stent graft explantation with preservation of fenestrated stent graft component to treat recalcitrant type II endoleak. J Vasc Surg Cases Innov Tech.

[17] Wang S.K., Fajardo A., Motaganahalli R.L., Gupta A.K. (2018). Successful treatment of an infected Zenith fenestrated endograft without explantation. Vasc Endovascular Surg.

[18] Oderich G.S., Bower T.C., Hofer J., Kalra M., Duncan A.A., Wilson J.W. (2011). In situ rifampin-soaked grafts with omental coverage and antibiotic suppression are durable with low reinfection rates in patients with aortic graft enteric erosion or fistula. J Vasc Surg.

[19] Charlton-Ouw K.M., Sandhu H., Huang G., Leake S.S., Miller C.C., Estrera A.L. (2014). Reinfection after resection and revascularization of infected infrarenal abdominal aortic grafts. J Vasc Surg.

